# Identification of mutations in SARS-CoV-2 PCR primer regions

**DOI:** 10.1038/s41598-022-21953-3

**Published:** 2022-11-04

**Authors:** Anikó Mentes, Krisztián Papp, Dávid Visontai, József Stéger, VEO Technical Working Group, István Csabai, Krisztián Papp, Dávid Visontai, József Stéger, Guy Cochrane, Nadim Rahman, Carla Cummins, David Yu Yuan, Sandeep Selvakumar, Milena Mansurova, Colman O’Cathail, Alexey Sokolov, Ross Thorne, Marion Koopmans, David Nieuwenhuijse, Bas Oude-Munnink, Nathalie Worp, Clara Amid, István Csabai, Anna Medgyes-Horváth, Orsolya Anna Pipek

**Affiliations:** 1 Department of Physics of Complex Systems, Eötvös Loránd University, Budapest, Hungary; 2 European Molecular Biology Laboratory, European Bioinformatics Institute, Wellcome Genome Campus, Hinxton, Cambridge CB10 1SD UK; 3 Department of Viroscience, Erasmus University Medical Center, Rotterdam, The Netherlands

**Keywords:** Genome informatics, Bioinformatics, Sequencing

## Abstract

Due to the constantly increasing number of mutations in the SARS-CoV-2 genome, concerns have emerged over the possibility of decreased diagnostic accuracy of reverse transcription-polymerase chain reaction (RT-PCR), the gold standard diagnostic test for SARS-CoV-2. We propose an analysis pipeline to discover genomic variations overlapping the target regions of commonly used PCR primer sets. We provide the list of these mutations in a publicly available format based on a dataset of more than 1.2 million SARS-CoV-2 samples. Our approach distinguishes among mutations possibly having a damaging impact on PCR efficiency and ones anticipated to be neutral in this sense. Samples are categorized as “prone to misclassification” vs. “likely to be correctly detected” by a given PCR primer set based on the estimated effect of mutations present. Samples susceptible to misclassification are generally present at a daily rate of 2% or lower, although particular primer sets seem to have compromised performance when detecting Omicron samples. As different variant strains may temporarily gain dominance in the worldwide SARS-CoV-2 viral population, the efficiency of a particular PCR primer set may change over time, therefore constant monitoring of variations in primer target regions is highly recommended.

## Introduction

The COVID-19 pandemic has been going on for over 2 years, and PCR-based diagnostics is still the major tool for the identification of SARS-CoV-2 infected people by successful amplification of the virus from nasopharyngeal or oropharyngeal swabs. The average mutation rate of the SARS-CoV-2 genome is estimated to be 1.05 × 10^–3^ to 1.26 × 10^–3^ nucleotide substitutions/site/year^1,2^, which is in the same order of magnitude as that of SARS-CoV^3^. In contrast, the human genome-wide mutation rate is approximately 0.5 × 10^−9^ per base pair per year^4^. Given the highly mutation-prone property of viruses, genetic variations in the viral genome in the primer/probe-binding regions can lead to false-negative results during polymerase chain reaction (PCR) detection^5^. Diagnostic primer/probe alignments have been performed by laboratories with a limited number of viral sequences in the early stages of the pandemic and some mismatches have been reported^6,7^, which may lead to false-negative results^8^. Since then, numerous publications have reported instances of false-negative diagnoses of COVID-19^9–12^. Due to the great clinical relevance of these mutations, there is a requirement to monitor primer/probe variations using sequences from virus isolates worldwide.

Throughout the analyses and discussions of this manuscript, we intend to adhere to a rigorous terminology to avoid confusion. We define a “primer system” as the collection of the forward and reverse primers (and whenever applicable, the probe) designed for the amplification and detection of a single genomic region during PCR. We refer to the parts of the genome where the forward and reverse primers (along with the probe, when relevant) bind as “target regions” (TRs). Thus, a given primer system has two or three TRs in the virus genome, depending on the exact scheme of the laboratory procedure. In order to reliably detect the presence of SARS-CoV-2 in a sample, it is advantageous to amplify multiple parts of its genome to avoid possible false-negative cases. Thus, many developers employ multiple primer systems in their PCR tests as a fail-safe. We term the assortment of primer systems designed by the same developer and used concurrently in a single test a “primer set”.

The impact of a variant on the efficacy of PCR tests can be influenced by various factors. The most known components determining the consequence of a variant are its specific genomic location within the TR^13,14^, and the total number of mutations overlapping the TR^15,16^. An additional aspect to be considered is the type of variant (point mutation or insertion/deletion), and if the former, whether it is a transversion or a transition^17,18^.

The potential complication presented by targeting highly polymorphic regions of the virus genome has been previously addressed by Davi et al.^19^ with the suggested solution of designing a primer set in silico optimized to target well-conserved sections instead. However, the study did not investigate either the actual number of variations affecting the TRs of previously developed primer sets or their ability to truly hamper the PCR process.

Here we aim to create a workflow to detect genomic variations compared to the original Wuhan reference sequence (NC_045512.2) that overlap the TRs of commonly used PCR primer sets (Table 1; full sequences, location and additional information of primers and probes are available on GitHub at the repository github.com/csabaiBio/coveo_pcr_primers2021). To this end, we use the CoVEO database that assembles data of more than 1.2 million good-quality SARS-CoV-2 samples sequenced from the start of 2021 to 6th of April 2022, originally uploaded to the COVID-19 Data Portal (https://www.covid19dataportal.org^32^).

**Table 1:** SARS-CoV-2 PCR primer sets analyzed in this study.

Assay name	Source/country	Target gene(s) (total number of TRs)	Technology
Chan-set^20^	University of Hong Kong (HKU)/Queen Elizabeth Hospital (QMH), China	RdRp, N, S (3)	Taqman
Chu-set^21^	Li Ka Shing Faculty of Medicine, The University of Hong Kong (HKU), China	ORF1ab, N (2)	Taqman
Corman-set^6^	Charité Hospital, Germany	RdRp, N, E (3)	Taqman
Davi-set^19^	Federal University of Rio Grande do Norte (UFRN), Brazil	ORF1ab, S (9)	Taqman
DMSC-set^22^	Ministry of Public Health (MOPH), Thailand	N (1)	Taqman
Huang-set^23^	Wuhan Jinyintan Hospital (Jin Hos), China	E (1)	Taqman
IP-set^22^	Institut Pasteur (IP), France	RdRp (2)	Taqman
Lu-set^8^	Centers for Disease Control and Prevention, USA (CDC-US)	N (3)	Taqman
Mollaei-set^24^	Kerman University of Medical Sciences (KMU)/Pasteur Institute of Iran (IPI), Iran	ORF1ab, RdRp, N, E, S (5)	Traditional
Niu-set^25^	Chinese Center for Disease Control and Prevention, China (CDC-China)	ORF1ab, RdRp, N, E (4)	Taqman
Sarkar-set^26^	Jashore University of Science and Technology (JUST), Bangladesh	RdRp, N, E, S (4)	SYBR Green
Shirato-set^27^	National Institute of Infectious Diseases (NIID), Japan	N (1)	Taqman
Tombuloglu-set^28^	Institute for Research and Medical Consultations (IRMC), Saudi Arabia	RdRp, E (2)	Taqman
Won-set^29^	Institute for Basic Science (IBS)/Seoul National University (SNU), South Korea	RdRp, N, E, S (9)	SYBR Green
Yip-set^30^	Queen Mary Hospital (QMH)/The Chinese University of Hong Kong (HKSAR), Hong Kong	ORF1ab (1)	SYBR Green
Young-set^31^	National Centre for Infectious Diseases (NCID), Singapore	ORF1ab, N, S (3)	Taqman

Primer set names are based on the first author’s last name of the reference.

The CoVEO database stores, in a coherent and searchable manner, the genomic variations of sequenced SARS-CoV-2 samples, which were produced by a freely accessible standardized variant calling workflow (see “Methods”). In order to verify our results on another dataset, the GISAID database (Global Initiative on Sharing All Influenza Data, https://www.gisaid.org^33^) was utilized to collect genomic variations of the SARS-CoV-2 genomes that could be processed with the same post-processing workflow that was used on the CoVEO database.

One of our main goals is to provide a comprehensive, raw list of mutations overlapping PCR primer TRs in the investigated samples, which can be further filtered based on individual scientific needs when investigating the possible effects of mutations on PCR performance or designing new PCR primer sets.

In this work, based on literature, we differentiate between mutations likely to affect the efficiency of PCR and ones predicted to be harmless in this sense. Our further goal is to perform an analysis that can forewarn the possibility of specific primer sets becoming obsolete due to emerging mutations in the virus genome.

## Results

### Ratio of samples affected by mutations in different primer system TRs

A raw list of mutations overlapping PCR primer TRs in the investigated samples is uploaded to a GitHub repository at github.com/csabaiBio/coveo_pcr_primers2021. For details on mutation filtering criteria, see “Methods”.

A total of 1,253,364 good-quality SARS-CoV-2 genomic samples were analyzed from the CoVEO database (see “Methods”) collected from the 1st of January, 2021 to the 6th of April, 2022 (see Supplementary Fig. 1). Most of these samples were Alpha or Delta variants, while the proportion of other VOCs (Variants of Concern) was significantly lower. Samples that could not be unambiguously categorized to WHO-designated lineages or were classified to a lineage other than Alpha, Beta, Gamma, Delta or Omicron were assigned the umbrella term “other variants” (Table 2). Even though at the end of 2021, the Omicron variant gained worldwide dominance, the relatively low number of Omicron samples in our dataset is due to inconclusive results of lineage designation by the preprocessing pipeline applied to samples prior to their upload to the CoVEO database. Thus many samples assigned to the “other variant” category from December of 2021 forward likely belong to the Omicron strain, but possess a reduced number of variant defining mutations.

**Table 2:** Total number of SARS-CoV-2 samples analyzed, and ratio of samples affected by a genomic variation in at least one investigated TR.

	All samples	Alpha	Beta	Gamma	Delta	Omicron	Other variants
Number of analyzed samples	1,253,364	210,308	3164	7859	678,190	40,105	313,738
Ratio of samples with variants in any of the investigated TRs (%)	96.78	99.04	95.45	98.84	99.35	99.63	89.29

**Figure 1:**
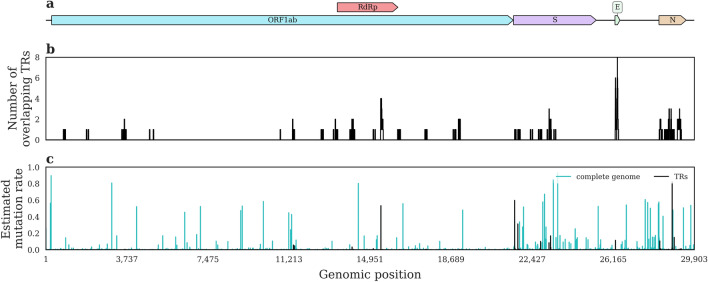
Overview of PCR primer TRs and average rate of mutations along the length of the SARS-CoV-2 genome. (**a**) SARS-CoV-2 isolate Wuhan-Hu-1, complete genome (NCBI ID of the fasta sequence: NC_045512) showing genes coding proteins located in ORF1ab (including RdRp), spike protein (S), envelope protein (E), and nucleocapsid protein (N). (**b**) Number of TRs overlapping a genomic position across all investigated primer sets. (**c**) Estimated mutation rate of a genomic position in the CoVEO database. For details, see “Methods”. For the estimated mutation rate of different genomic positions in samples belonging to various variant strains, see Supplementary Fig. 2.

We found reliable genetic variations in 1922 of all 2188 genomic positions overlapping the 141 primer or probe binding sites (TRs) in the investigated SARS-CoV-2 samples. In many cases, different primer sets target the same sections of the genome. For example, primer systems designed for the E gene of the genome necessarily share some of their TRs due to the short length of the gene (Fig. 1a,b). The E gene also has a low estimated mutation rate across all investigated samples (Fig. 1c), in line with basic intuition that primer systems are best designed to target relatively conserved regions of the genome. However, in different variant strains, different genomic regions tend to be mutated frequently (Supplementary Fig. 2). For instance, in Omicron samples, the generally rarely mutated E gene contains a TR which is affected in more than 50% of the cases.

Most of the mutations affecting the TRs were point mutations (with a slightly lower frequency of transitions (1758) than transversions (1827)), while the numbers of distinct deletions (148) and insertions (30) were significantly lower.

**Figure 2:**
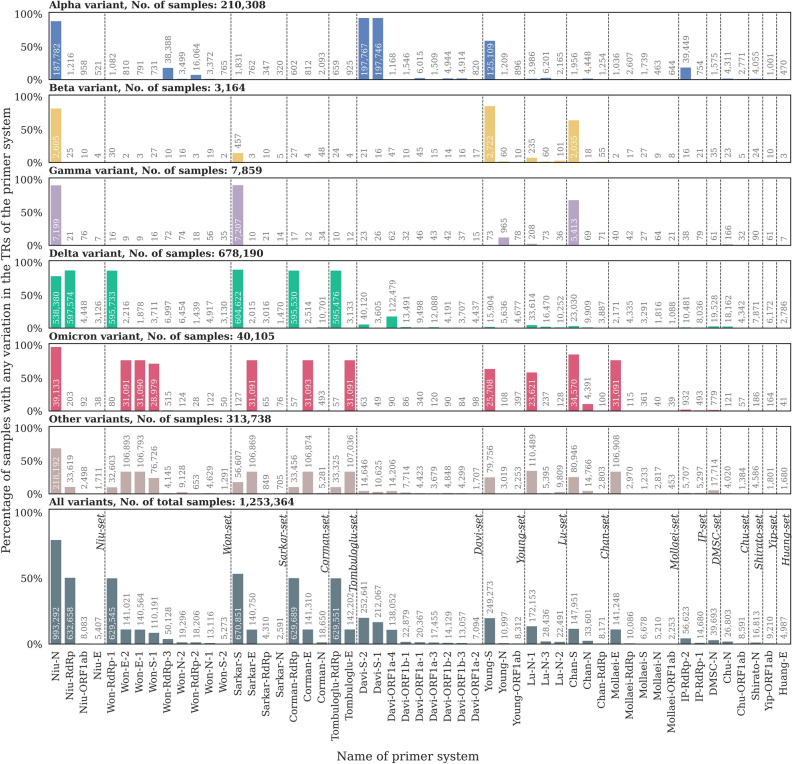
Percentage and number of samples with any mutations in the TRs of a given primer system, colored by WHO designation. Primer system names are based on the nomenclature: [first author last name]-[target gene name]-[id, when multiple primer systems target the same gene]. Samples with no variants in the given TRs are not shown.

The ratio of samples with any variants in the TR of a given primer system (any of its forward primer/probe/reverse primer regions) was calculated (Fig. 2, bottom panel). We found that even for the primer system targeting the seemingly most conserved genomic regions (Mollaei-ORF1ab), 2253 (< 0.2%) samples contained at least a single mutation in the TRs. On the other hand, the ratio of samples affected by at least one variant is below 15% for 44 of the 53 investigated primer systems. In the TRs of the remaining 9 primer systems a considerable fraction of the samples had at least one variant: almost 80% of the samples contained a mutation in the TRs of primer system Niu-N; about 50% of samples had a variant in the TRs of primer systems Niu-RdRp, Won-RdRp-1, Corman-RdRp, Tombuloglu-RdRp, and Sarkar-S, furthermore around 17–20% of samples were mutated in the TRs of primer system Davi-S-2, Davi-S-1 and Young-S.

Different variant strains show highly diverse mutational patterns in various primer systems (Fig. 2, top six panels). While many samples tend to have a mutated TR in the Niu-N primer system independent of their lineage, the TRs of many primer systems are almost exclusively mutated in samples of a specific variant (e.g. Davi-S-1 and Davi-S-2 systems are mainly affected in Alpha samples, the TRs of the Young-S system are usually mutated in Alpha, Beta and Omicron samples, the TRs of the Sarkar-E system are mainly altered in Omicron samples, etc.).

This result suggests that the performance of a given primer set largely depends on the specific genomic characteristics of the presently circulating most dominant lineages. Thus, PCR efficacy should be dynamically reevaluated throughout the course of the pandemic.

### Possible effects of mutations on PCR amplification

**Figure 3:**
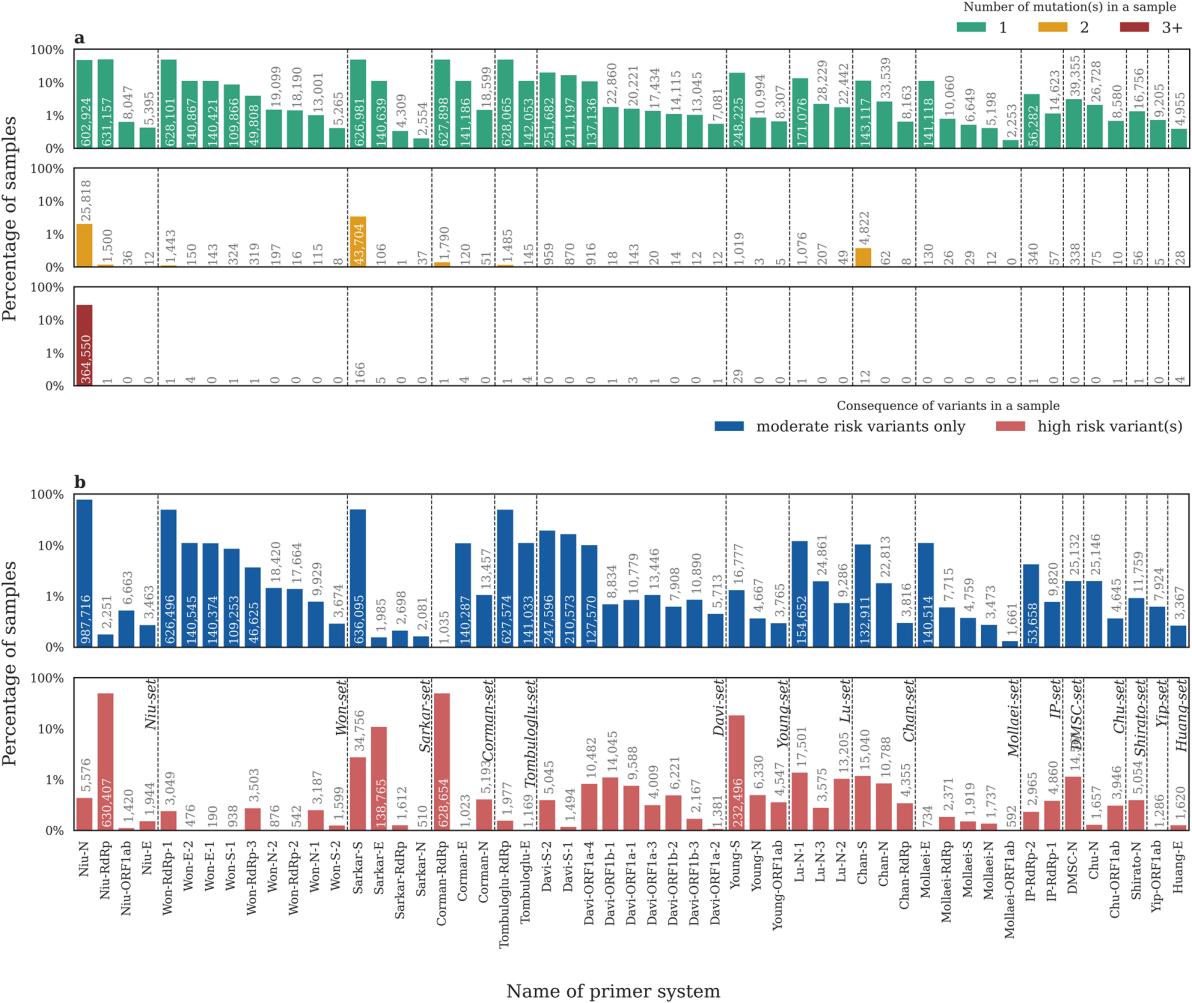
Number of mutations and their possible effect on PCR amplification. (For another version of the figure with linear vertical axes, see Supplementary Fig. 3. Supplementary Figs. 4–9 contain the same results separately for different variant strains). (**a**) The percentage and number of samples with one (green bars), two (yellow bars) and three or more (red bars) variants in the TRs of different primer systems. (**b**) The percentage and number of samples with variants in the TRs of different primer systems. Samples that contain a variant in at least one “high risk” position in the TRs of the given primer system are marked with red, other samples having only “moderate risk” mutations in the given TRs are presented in blue. For further details on mutation classification, see “Methods”. Primer system names are based on the nomenclature: [first author last name]-[target gene name]-[id, when multiple primer systems target the same gene]. Samples with no variants in the given TRs are not shown.

We calculated the ratio of samples with a single, two, and three or more genomic variations in the TRs of a given primer system. As shown in Fig. 3, most of the affected samples have only a single variant position (Fig. 3a green bars) over the TRs. Nevertheless, there are a few samples for most primer systems with two or more variations present in the TRs (Fig. 3a yellow and red bars), but their number is generally below 5000, accounting for less than 0.4% of all samples. A notable exception is the TRs of the Niu-N primer system, in which more than 364,550 samples (about 30% of all samples) contained at least three mutations, with one of the samples presenting seven variant positions. Another primer system with TRs commonly containing multiple mutations is the Sarkar-S system, for which 43,870 samples had more than one genomic variant. On the other hand, none of the samples had multiple mutations in the TRs of the Mollaei-ORF1ab system and the number of samples (2253) containing a single mutation was also exceptionally low.

As a next step, we examined the type of the detected variants and their location in the TRs of different primer systems and categorized them as either “high risk” or “moderate risk” mutations (see “Methods” for details). 0.015% to 50.30% of the samples contain high risk mutations for a particular primer set. The distribution of samples with variants belonging to different risk-categories is presented in Fig. 3b. Most of the samples that had any mutations in the TRs of any given primer system contained only variants with no drastic effect on PCR efficiency based on their location. For example, the highly mutation-prone TRs of the Niu-N primer system usually contain variants at moderately risky positions which are unlikely to disrupt the PCR process. In contrast, the TRs of two primer systems (Niu-RdRp and Corman-RdRp) are mutated in high risk positions in many samples, comprising around 50% of the total samples analyzed.

Based on our results, the most common high and moderate risk mutations that were identifiable in the majority of samples are listed in Table 3.

**Table 3:** Summary of the most frequent mutations in the TRs of investigated PCR primer systems.

Primer	Mutation	Mutation consequence	Defining mutation in VOC	Ratio of mutated samples in the CoVEO database (%)	Ratio of mutated samples by WHO designation (*)
Sarkar-S-F^M^	SNP: C21618G	S: T19R	Delta	50.52	Delta (89.13%), other variant (9.15%)
Corman-RdRp-F^H^, Niu-RdRp-F^H^, Tombuloglu-RdRp-F^M^, Won-RdRp-1-F^M^	SNP: G15451A	Synonymous	–	50.03	Delta (87.79%), other variant (10.04%), Beta (< 1%), Omicron (< 1%), Alpha (< 1%), Gamma (< 1%)
Niu-N-F^M^	SNP: G28881T	N: R203M	Delta	44.83	Delta (79.16%), other variant (7.98%), Alpha (< 1%)
Niu-N-F^H^	“AAC”-triplet: G28881A, G28882A, G28883C	N: R203K, G204R	Alpha, Omicron	29.07	Omicron (90.62%), Gamma (90.19%), Alpha (84.93%), other variant (45.36%), Delta (< 1%)
Young-S-F^H^	Deletion: ATACATG21764A	S: H69_V70del	Alpha, Omicron	17.40	Omicron (64.04%), Alpha (58.33%), other variant (22.17%), Delta (< 1%)
Davi-S-1-P^M^, Davi-S-2-P^M^	SNP: C23271A	S: A570D	Alpha	16.52	Alpha (93.98%), other variant (2.99%), Delta (< 1%)
Niu-N-R^M^	SNP: C28977T	N: S235F	Alpha	12.88	Alpha (74.97%), other variant (1.19%), Gamma (< 1%), Delta (< 1%)
Sarkar-E-F^H^, Corman-E-F^M^, Mollaei-E-F^M^, Tombuloglu-E-F^M^, Won-E-1-F^M^, Won-E-2-F^M^	SNP: C26270T	E: T9I	Omicron	11.06	Omicron (77.52%), other variant (33.93%), Alpha (< 1%), Gamma (< 1%), Delta (< 1%), Beta (< 1%)
Lu-N-1-probe^M^	SNP: C28311T	N: P13L	Omicron	10.20	Omicron (58.86%), other variant (33.0%), Beta (< 1%), Gamma (< 1%), Delta (< 1%), Alpha (< 1%)

For more details, regarding mutation position and estimated effect in the different primers, see Supplementary Table 1.

Primer names are based on the nomenclature: [first author last name]-[target gene name]-[id, when multiple primer systems target the same gene]-[type of oligo: forward (F), reverse (R) or probe (P)]. “M” marks the primers where the variant was defined as a moderate-risk mutation; “H” marks the primers if the variant was defined as a high risk mutation. Mutation names are based on the nomenclature: [reference base][genomic position of the start of the variant][alternate non-reference base]. Asterisk: ratio of samples which contain the mutation in a given WHO designation. In the fourth column, those VOCs are listed in which the given mutation appears as a defining one. Lineages with no mutated samples are not listed.

*SNP* single-nucleotide polymorphism.

### Potential false-negative results due to misclassification

Since diagnostic COVID-19 tests generally aim to amplify several gene regions simultaneously, thus employing primer sets of multiple primer systems, we investigated whether there are samples with damaged TRs (see “Methods” for definition) in multiple primer systems of specific primer sets. We differentiated between samples having a “slight change of misclassification” and samples “susceptible to misclassification” with a primer set based on the number and ratio of damaged TRs in the primer systems of the given set. Samples with no damaged TRs in the set and sufficient sequencing depth for all of them were regarded as having “no reasonable chance of misclassification” (see “Methods” for details).

**Figure 4:**
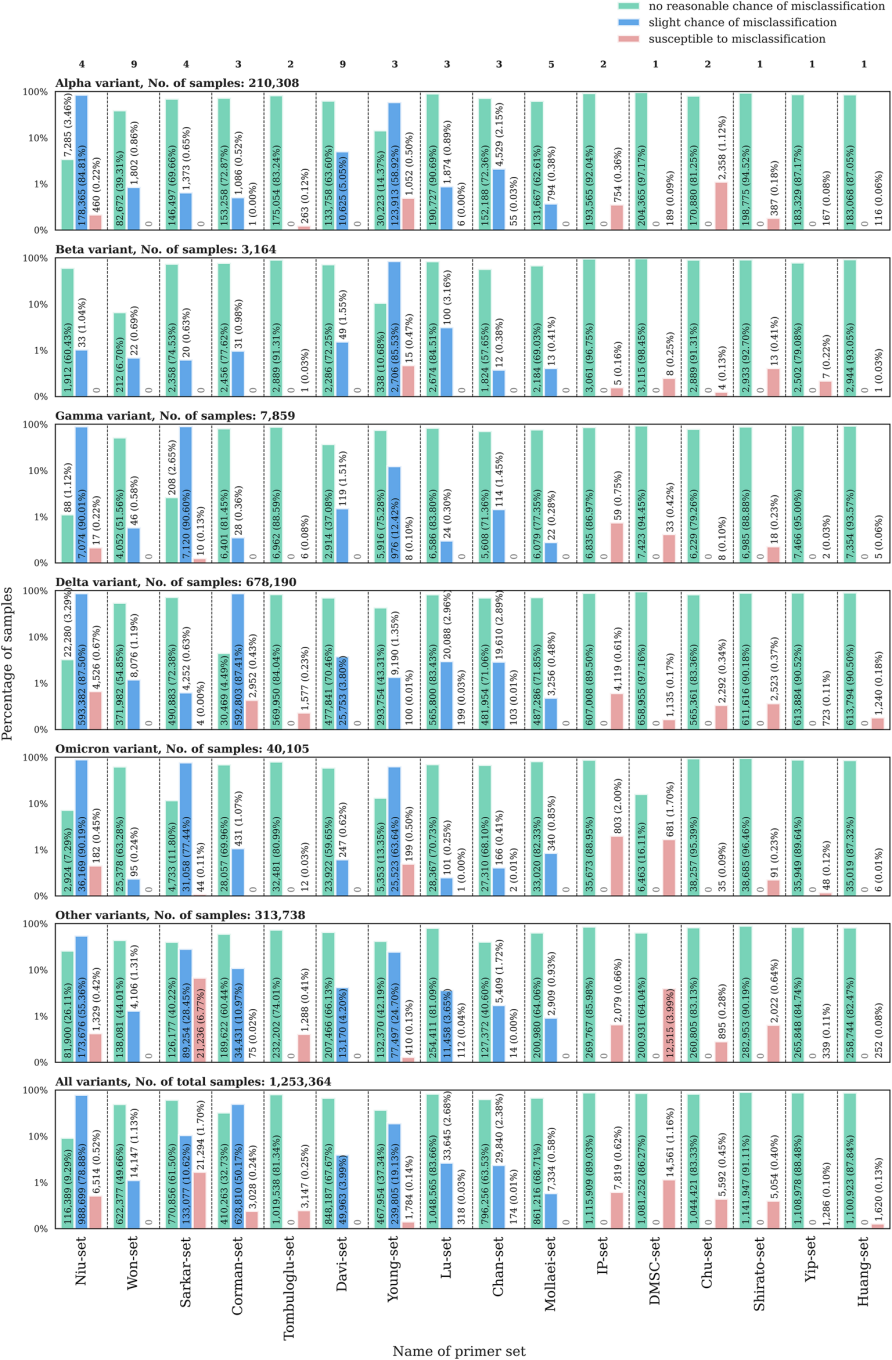
Percentage and number of samples having no reasonable chance (light green) or a slight chance of misclassification (light blue) or being susceptible to (light red) misclassification by different primer sets. Numbers on top indicate the number of primer systems present in a given primer set. Primer-set names are based on the nomenclature: [first author last name]-[set]. Ambiguous samples with unsatisfactory coverage in TRs are not shown. For a modified version of the figure with linear vertical axes, see Supplementary Fig. 10.

A relatively large number of samples had a slight chance of misclassification with the Niu-, Corman- or Young-sets, with respectively only 9.29%, 32.73% and 37.34% of them having evidence of absolutely no damaged TRs (Fig. 4).

Nevertheless, there is only a negligible number of samples (with a maximum ratio of 1.70% for the Sarkar-set) susceptible to misclassification with any of the investigated primer sets, and in most cases, only very few TRs of a primer set are damaged simultaneously in each sample. Based on these observations, for most primer sets, a dominant part (50.79–91.11%) of the investigated samples could be reliably detected as positive ones if partially inconclusive results are not rejected automatically by the test protocol (i.e., if a primer set consists of three primer systems, and among them, one is damaged, the result of the PCR is not automatically considered as negative).

An important additional insight is that the ratio of ambiguous samples (not shown in Fig. 4) with no satisfactory coverage across all TRs for a definite categorization vary greatly for different primer sets. This is partly explained by the fact that the number of primer systems employed by a given set is also highly variable and statistically there is a smaller chance to obtain a sample with high enough coverage in all TR positions for 9 primer systems (e.g. for the Won-set 49.21% of all samples were ambiguous) than it is for a single one (e.g. for the Shirato-set the same ratio was 8.49%). On the other hand, some primer sets are notable exceptions to this trend. For example the Davi-set, also containing 9 primer systems, had inconclusive results for only 28.34% of the samples. On the contrary, for the Young-set with only 3 primer systems 43.39% of the samples were ambiguous.

It is also worth noting that primer sets with an overall low proportion of samples susceptible to misclassification can have an increased chance of failure in cohorts of samples belonging to a specific variant. For example, the IP-set showed an appeasing 0.62% for the proportion of samples susceptible for misclassification across all sample groups, but particularly for Omicron samples this ratio increased to 2.0%. More prominently, for the Sarkar- and DMSC-sets the percentage of samples susceptible to misclassification in the whole dataset was 1.70% and 1.16% respectively, while specifically for the “other variant” category, these ratios increased to 6.77% and 3.99%, respectively. Many of these presumably problematic samples assigned to the “other variant” group are suspected to be in fact Omicron samples with ambiguous lineage designation results (for details, see the next section).

These results suggest that to truly minimize the number of samples susceptible to misclassification, it can be beneficial to simultaneously use three or more primer systems within a single PCR test. This way, even with a damaged TR, more than 50% of the employed primer systems would yield a positive test result. Notably, primer sets with at least 5 primer systems (Won-set, Davi-set, Mollaei-set) were extremely unlikely to misclassify samples due to mutations present in the TRs (see the lack of light red columns on Fig. 4, bottom panel), in fact, none of the samples were deemed susceptible to misclassification with these three sets.

Additionally, given that primer sets perform differently across variant groups, it is important to continuously survey the ratio of samples prone to misclassification to determine whether the given primer set is suitable for the detection of SARS-CoV-2 samples of the presently spreading lineage.

### Ratio of samples having a slight chance of or being susceptible to misclassification over time

**Figure 5:**
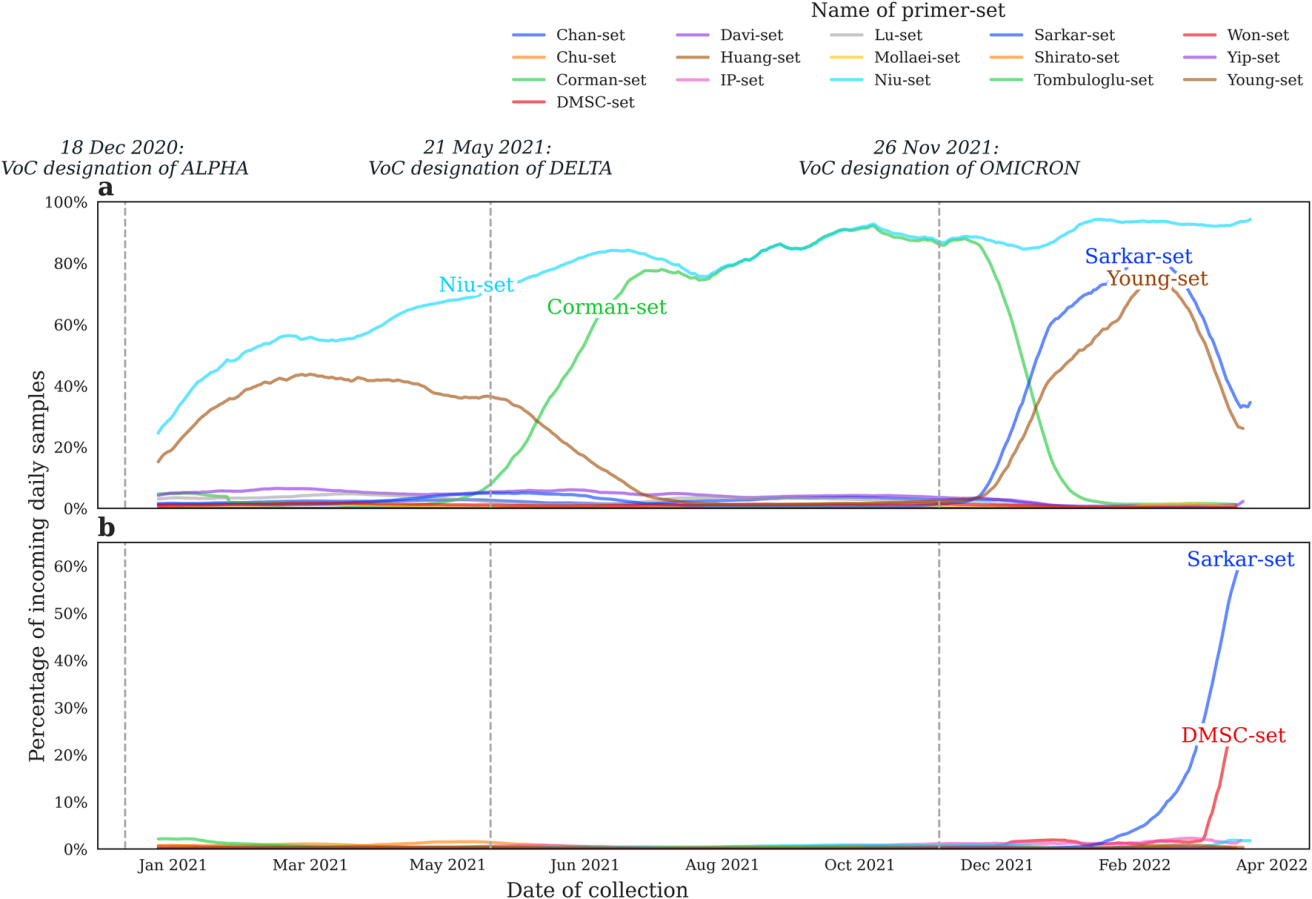
Percentage of samples (**a**) having a slight chance of or (**b**) being susceptible to misclassification with different primer sets over time (30-day rolling average) (for a zoomed-in version of the figure, see Supplementary Fig. 11).

It is also a matter of concern to monitor the relative occurrence of variants on the TRs of different primer systems over time to predict if a primer set is at danger of becoming obsolete as new strains of the virus emerge. The ratio of samples having a slight chance of (Fig. 5a) and being susceptible to (Fig. 5b) misclassification was calculated over time using a 30-day rolling average method. For a zoomed-in version of the lower panel, see Supplementary Fig. 11.

Most of the primer sets analyzed in this work (with the exceptions of the Davi-, Sarkar- and Tombuloglu-sets) were designed in 2020 at the beginning of the pandemic, with only a few SARS-CoV-2 genomes available, hence the mutational patterns of the more recent Alpha and Delta lineages were inaccessible at the time.

With the appearance of the Alpha variant in early 2021, the number of samples with at least one damaged TR of the Niu- and the Young-sets increased, due to mutations R203K, G204R on the N gene (affecting the Niu-set TRs) and HV69_70del on the S gene (overlapping a Young-set TR). Around June, with the emergence of the Delta variant, the mutation that damaged the TRs of the Young-set (S: HV69_70del) disappeared from the dominant portion of the samples, as Delta variants lack this mutation. At the same time, a new synonymous mutation (G15451A) appeared in one of the TRs of the Corman-set, causing the ratio of samples having a slight chance of misclassification with this primer set to increase, and also raising the number of samples having a slight chance of misclassification with the Niu-set, compensating for the disappearance of N: R203K and N: G204R in Delta samples. This trend seems to be reversing since the widespread arrival of Omicron samples (that lack the above G15451A mutation), which, however did not have an effect on the ratio of samples having a slight chance of misclassification with the Niu-set, due to the revival of variants N: R203K and N: G204R in Omicron samples. Additionally, the TRs of the Sarkar- and Young-sets seemed to be gaining damaging mutations in Omicron samples, thus samples having a slight chance of misclassification with these primer sets were getting more frequent since November of 2021. This was due to the renewed accumulation of deletion HV69_70del on the S gene (affecting one TR of the Young-set) and T9I on the E gene (affecting one TR of the Sarkar-set) of early Omicron samples. However, recently there has been a reduction in the numbers of samples with a slight chance of misclassification for both sets, albeit for completely different reasons. The deletion (S: HV69_70del) damaging one of the TRs of the Young-set is absent from Omicron BA.2 samples, which variant took dominance over the previous Omicron BA.1 strain around February–March of 2022.

On the other hand, the E: T9I mutation is consistently present in Omicron variants, thus the decrease in the ratio of samples with a slight chance of misclassification with the Sarkar-set is not explained by the disappearance of a mutation. On the contrary, along with the E: T9I mutation, Omicron BA.2 samples acquire the LPP24_26del deletion on their S protein, rendering the TRs of two primer systems of the Sarkar-set damaged. Thus samples with both of these mutations are considered susceptible to misclassification with the set, as at least half of its altogether four primer systems have damaged TRs. This causes the increasing trend for the Sarkar-set apparent on Fig. 5b, starting from February, 2022, and also the decreasing tendency around the same time on Fig. 5a.

The number of samples susceptible to misclassification is also increasing since December, 2021 (see Supplementary Fig. 11 for a more detailed graph) for the DMSC-set, as the ERS31_33del deletion on the N gene became widespread with the advance of Omicron samples. Given that this set employs only one primer system, a single high risk mutation in its TRs causes samples to immediately become susceptible to misclassification.

Other than the above described trends initiated by the appearance of the Omicron variant, the daily ratio of samples susceptible to misclassification remains under 3% for the whole timeline for all remaining primer sets. A few temporary peaks can be observed (Supplementary Fig. 11) for the Tombuloglu-, Chu- and Shirato-sets, but these are short-lived events and affect only a limited percentage of the samples.

It is important to note that either the spread of a new variant or simply the emergence of a damaging mutation within the dominant strain might drastically increase the number of samples prone to misclassification for any given primer set. Thus, it is essential to continuously monitor genomic variations overlapping the TRs of primer sets used in routine diagnostics. This is especially true, since many of the damaging mutations affecting primer TRs are in fact lineage defining ones, inherently putting many samples at risk of possible misclassification. To this end, we set up a regularly updated online platform which is able to monitor the daily rate of samples having a slight chance of and being susceptible to misclassification at https://k8plex-veo.vo.elte.hu/shiny/2/, under the left-hand side menu item “PCR primers”.

### Comparison with the GISAID database

We compared our results with genomic variants found in SARS-CoV-2 samples from the GISAID (http://www.gisaid.org^33^) database collected in the same time period as our original sample set, where a total of 8,368,941 samples (Number of samples classified by WHO-lineages: Alpha: 901,802, Beta: 311,710, Gamma: 407,750, Delta: 3,991,796, Omicron: 2,455,887) were analyzed. We found genetic variants in all 2188 genomic positions mapped to 141 primer or probe TRs in the investigated samples. We found that the ratio of GISAID samples containing either mutations of any kind, moderate risk mutations or high risk mutations in the TRs was similar to that of in the CoVEO database for all analyzed primer systems. The most frequent mutations overlapping the TRs in the CoVEO database are also present in the GISAID database with a similar frequency of affected samples (C21618G: 48.55%, G15451A: 48.25%, G28881T: 48.84%, “AAC”-triplet: 44.77%, C23271A: 13.15%, C28977T: 13.14%, C26270T: 29.17%, C28311T: 29.23%, ATACATG21764A: 19.33%) (for comparison see Table 3 and Supplementary Table 2). Additionally, in GISAID samples some frequent mutations predominantly affecting Omicron samples were found, which, given the relatively low number of Omicron samples in our dataset, were not identified as high-frequency variants in the CoVEO database. These mutations are also listed in Supplementary Table 2. When analyzing GISAID samples over time, we found that samples susceptible to misclassification were generally present at a daily rate of 4% or lower. On the other hand, the daily ratio of samples having a slight chance of misclassification with a certain primer set could reach almost 100%, similarly to our results on the CoVEO database. Additionally, mutations causing the decrease in performance of the DMSC- and Sarkar-sets from the beginning of 2022 were also present with high frequency in Omicron samples of the GISAID database (E: T9I (99.15% of Omicron samples); S: LPP24_26del (70.85% of Omicron samples); N: ERS31_33del (85.91% of Omicron samples)).

The consistent results acquired across multiple databases suggest that the mutations observed in CoVEO samples overlapping the TRs are not due to sequencing artifacts or the by-products of the bioinformatical analysis pipeline, but are in fact true genomic variants occurring frequently and possibly affecting PCR test accuracy. This is also supported by the fact, that many of the identified mutations were indeed well-established, lineage defining variations. Even though the obtained results are in great agreement across data providers, it should be underlined that samples of the CoVEO database were processed with a single, standardized, publicly available workflow, while GISAID consensus sequences are generated individually by data uploaders. Moreover, the CoVEO database contains detailed information about genomic variants (sequencing depth, alternate allele frequency, alternate alleles by read orientation, etc.), which can be utilized to specifically filter variants based on different scientific research requirements.

## Discussion

This study comprehensively evaluated the genetic variability of 53 previously published SARS-CoV-2 diagnostic primer systems of 16 primer sets in PCR primer/probe-binding regions, including those recommended by the WHO. We found that the TRs of many of the investigated primers were prone to mutations in the analyzed samples, but further investigations were needed to determine if these variations had the potential to reduce PCR sensitivity in a clinical setting.

Zimmermann et al.^34^ highlighted the fact that experimental data does not necessarily follow the theoretical predictions, particularly with regard to the magnitude of the Ct shift with mismatches close to the 3′ end. Moreover, the specific nucleotide composition of these mismatches also seemed to play a role in determining PCR efficacy^35^. In some protocols^36^, the results of the PCR test are automatically deemed inconclusive (thus not positive) if even a single primer system of the primer set fails to suitably amplify its targeted genomic region, which may also influence the correct evaluation of the samples. Furthermore, a common practice to reduce both testing time and cost is to pool samples prior to the PCR procedure, which inherently considerably limits sensitivity^37^, thus could result in an increased susceptibility to errors caused by mutations in the TRs.

Given that both the number of variations in the TRs of the employed primers and their relative position to TR end sites can influence the efficacy of PCR reaction, we considered both of these factors in the investigated samples and assigned variants to be either high risk or moderate risk based on their relative position in a given TR. According to Bru et al.^13^, a single mutation can result in an underestimation of the gene copy number by up to 1000-fold. The number of mutations within a TR shows a negative correlation with the PCR amplification efficiency^16,38^. Mismatches at the 3′ end are known for their deleterious effect on PCR amplification, and even a single 3′ end mismatch can lead to a failed PCR reaction^39^. On the other hand, single mismatches, especially more than 5 bp away from the 3′ end, have only a moderate effect on PCR amplification and are unlikely to significantly affect the assay performance^5,18,35^.

Our results showed that most of the samples containing any variation in the TRs of a primer/probe generally had a single mutation, which is in most cases unlikely to drastically influence the effectiveness of the PCR process. However, we found that the most frequent SNP overlapping any of the TRs (G15451A, see Table 3) could be identified in more than half of the samples, mainly belonging to the Delta variant. This SNP was defined as high risk in two forward primers. Vogels et al.^40^ reported that this mutation was present in 100% of the samples they have tested. Regardless, there are samples with multiple variants in the TRs of some primers. The most common multiple variation (affecting the 5′ end of the Niu-N forward primer binding site) was the “AAC” triplet (Table 3), which was already described in several publications^40–46^, but the studies reported varying frequencies (13–37%) of the 'AAC' mutant in the GISAID samples they investigated. We also found that the His69_Val70del deletion of the Spike protein, overlapping the Young-S forward primer TR, was present in a relatively high proportion of the samples in the time range when the Alpha variant gained dominance worldwide. It has been previously demonstrated^47^ that this causes S-gene target failure on the TaqPath COVID‑19 PCR test (ThermoFisher). Two sublineages of the Omicron strain (BA.1, BA.3) also contain this deletion, which might cause a renewed reduction in PCR efficiency for the Young-set and the TaqPath kit^47^.

The CoVEO database used in this study provides the advantage of fast and straightforward mutation retrieval compared to databases containing only the consensus sequences of the samples. Even though the number of genomic variations occurring in the TRs of the investigated primer sets is generally low and the ratio of affected samples remains under 2%, a readily deployable pipeline for monitoring mutation frequency in the TRs is of utmost importance.

To improve COVID-19 diagnostic test efficiency and sensitivity, it is common practice to employ multiple primer systems in order to target multiple regions of the virus genome within a single PCR assay. We detected a relatively large number of samples that had at least one primer system within a primer set that had a damaged TR in the sequenced genome, defining these samples as having a slight chance of misclassification with the given assay. On the other hand, the number of samples that had damaged TRs for more than half of the primer systems in the set (samples “susceptible to misclassification”) was generally negligible for all investigated primer sets. This underlines the importance of using more than one target in diagnostic PCR tests already pointed out by previous studies^28,34^.

To monitor whether samples with high risk mutations in the TRs of the different primer sets are becoming more frequent in time, we plotted the fraction of samples having a slight chance of misclassification and being susceptible to misclassification. We found that the frequency of samples prone to misclassification was changing during the analyzed time period in strong correlation with the emergence of the different VoCs. This result highlights the need for constantly overseeing emerging mutations, especially in the case of the appearance of a new SARS-CoV-2 lineage. This way the primer sets used in clinical and commercial settings can be regularly reevaluated and updated if necessary.

Recent efforts in similar aspects have been made by aligning a limited number of viral sequences with primers/probes to look for mismatches^40–46,48^. Nayar et al.^48^ found that there is a growing number of mismatches, with an increase of 2% per month, and emerging mutations are highly specific to various geographic locations. Their previous statement is in agreement with estimations on the general mutation rate of the virus, and in addition to this observation, our results also suggest that the mutational landscape of a new VoC does not automatically contain the same variations as the previous VoCs, i.e. a new VoC does not necessarily emerge from a previous, widespread variant. Peñarrubia et al.^46^ found that about one-third of the genomes they tested included single mutations affecting the annealing of any PCR assay. Variations in the quarter of their investigated samples were considered high risk, whereas additional (less than ten percent) genomes presented low frequency single mutations that were predicted to yield no impact on sensitivity.

In conclusion, given the previously published data and the bioinformatic analysis performed in this study, currently, the known variability in the SARS-CoV-2 population has in most cases minimal or no impact on the sensitivity of existing molecular systems for virus detection. Notable exceptions are the DMSC- and Sarkar-sets, for which the number of samples susceptible to misclassification has drastically increased since the advance of Omicron samples. The majority of the commonly observed variants were not high risk ones (near the 3′ end of the TR/multiple mutations/indels) that could potentially disrupt the PCR process, but a few exceptions should be highlighted: one trinucleotide mutation (G28881A, G28882A, G28883C), one deletion (ATACATG21764A), and two SNPs in primer TRs near the 3′ end (G15451A, C26270T), which occurred with high frequency in the samples. Our results suggest that the detection of Alpha and Delta variants can be confidently performed with any of the investigated 16 primer sets. On the other hand, Omicron variants might be increasingly hard to identify with the use of the DMSC- and Sarkar-sets, but the performance of the remaining 14 primer sets was not compromised.

Our approach providing these results is unique in both the sense that we only included good-quality samples and high-confidence variants determined from raw sequencing data in our analysis instead of investigating consensus sequences; and in its comprehensive way of differentiating between harmless and possibly damaging mutations. Our work is aimed to draw attention to the need of constant surveillance of mutations affecting already existing and yet-to-be-developed primer sets. Nevertheless, due to the scarce access to primer and probe sequences used in commercial SARS-CoV-2 PCR tests, our results are inherently limited to the publicly available, but in practical settings rarely used primer sets.

However, it should be mentioned that viral genomes harboring mutations that are truly capable of escaping PCR amplification during clinical testing are unlikely to be submitted to sequencing later. Thus, it is possible that the reliable identification of these extremely high risk, but also incredibly low-frequency mutations would be impossible by analyzing sequencing data, given that due to their rarity, the mutated samples would not be confronted with a wide selection of available PCR primer sets.

## Methods

Through international effort, the Versatile Emerging infectious disease Observatory (VEO, http://www.veo-europe.eu) consortium analyses and interprets genomic data from SARS-CoV-2 sequencing samples as one of its subprojects. Throughout its standardized pipeline, variants of the sequenced samples submitted to the European COVID-19 Data Portal (http://www.covid19dataportal.org^32^) are identified and stored in VCF files, the results of which are then loaded into a PostgreSQL database, named CoVEO. This data is unique in the sense that besides the commonly available consensus sequences (for example in the GISAID database, http://www.gisaid.org^33^), the raw sequencing data of the samples is also accessible. This allows for direct filtering of genomic positions based on sequencing depth and alternate allele frequency.

The standardized pipelines for variant calling are publicly available on GitHub^49,50^.

In our analyses only those of the total 1,642,779 samples of the CoVEO database were included that were collected between 1st January 2021 and 6th April 2022 and had an estimated N-content of no more than 10% (estimated N-content was defined as the ratio of genomic positions in a sample with a sequencing depth of less than 10). This filtering step resulted in 1,253,364 good-quality samples.

In order to restrict our analyses to highly reliable variants, genomic positions where the sequencing depth did not reach 100 and/or the alternate allele frequency was below 0.9 were discarded.

Mutation rate at each genomic position (Fig. 1c) was calculated by dividing the number of samples with a high-confidence (see above) mutation at the given position by the total number of samples with a coverage of 100 or more in the same position.

Sequences and data for 53 (traditional and RT-Q) PCR SARS-CoV-2 detection primer systems, belonging to 16 different primer sets were collected from the literature^6,8,19–21,23–31^ or obtained from WHO^22^. In this study, primer system names are based on the nomenclature: [first author last name]-[target gene name]-[id, when multiple primer systems target the same gene]. The sequences of primers and probes were aligned to the Wuhan reference genome of SARS-CoV-2 (NC_045512.2) to determine their TRs within the genome using BLAST^51^. Only those mutations were considered that overlapped the TRs of the above primer systems.

Previous explorations of PCR efficacy^5,18,35,39^ suggest that variations at the 3′ end of the TR of either the forward or reverse primer are more prone to hinder the PCR reaction than mutations in other parts of the TRs. In contrast, variants in the middle of the probe TR are more likely to reduce detection efficiency than near-end mutations^52,53^. Therefore, we designated all insertions and deletions, along with mismatches that occur in the first 5 positions of the 3′ end of the forward and backward primer TRs or the middle of the probe TR (5 base pairs inward from the two ends) as “high risk” mutations and assigned “moderate risk” to the rest of the variants.

It has also been experimentally demonstrated that an increased number of mutations (of any kind) in the TR of the forward/reverse primers or the probe can reduce duplex stability, thus impairing amplification and detection of the targeted genome regions. For primers with an approximate base length of 30, two to four internal (non-3′ terminal) mismatches had no significant effect on RT-PCR, however, 6 to 8 mismatches reduced the PCR product yield by approximately 22–100-fold respectively^15^. Samples with viral genomes that harbor either a high risk mutation in the TRs of a specific primer system and/or possess an increased number of variations (of any kind) in a single TR of the same primer system are at risk of escaping PCR amplification. To that end, primer sets usually consist of multiple primer systems to decrease the probability of a false-negative result. Theoretically, a sample containing the genome of SARS-CoV-2 will only be categorized as negative if all the primer systems of the applied test fail to amplify and/or detect their targeted genome regions. Recently Laine et al.^54^ showed that samples having high risk mutations (a short 3 bp deletion and three subsequent mismatches) in the TR of the N gene, resulted in no signal for this primer system, however, the other primer system (targeting ORF1ab) of the primer set showed prominent signal, suggesting the presence of the SARS-CoV-2 genome in the sample. Thus, we consider samples “susceptible to misclassification” by a given primer set if more than 50% of the primer systems of the set have TRs that are damaged by mutations. A TR is defined to be damaged if at least a single high risk mutation or a minimum of 3 mutations of any kind are present in it. Samples with at least one damaged TR in the primer systems of the given primer set are regarded as having a “slight chance of misclassification” if no more than 50% of the primer systems of the given set have damaged TRs. Samples that had a coverage of 100 or more in all the genomic positions overlapping any of the TRs of a given primer set and none of these TRs were proven to be damaged were regarded as having “no reasonable chance of misclassification”. We emphasize that this is an extremely stringent categorization and is aimed at monitoring samples with even the slimmest probability of escaping PCR amplification. It should also be noted that the above three categories include only those samples for which a credible proof of either a minimum of a single damaged TR or of absolutely no damaged TRs exists. Thus, samples with ambiguous results (i.e. with no proof of a damaged TR but with insufficient coverage in any of them) are not considered.

## Supplementary Information

Supplementary Information.

## Data Availability

The datasets generated and/or analysed during the current study are available in the coveo_pcr_primers2021 repository, https://github.com/csabaiBio/coveo_pcr_primers2021.
