# Supplementary material for: Identification of mutations in SARS-CoV-2 PCR primer regions
Source: Sci Rep. 2022 Nov 4;12:18651. doi: 10.1038/s41598-022-21953-3 (PMC9636223; doi:10.1038/s41598-022-21953-3)
Supplement: Supplementary file 1 — Supplementary Information. [file 41598_2022_21953_MOESM1_ESM.pdf]

Supplementary Information

Identification of mutations in SARS-CoV-2 PCR primer regions

Anikó Mentes, Krisztián Papp, Dávid Visontai, József Stéger, VEO Technical Working Group, István Csabai, Anna Medgyes-Horváth, Orsolya Anna Pipek

This file includes:

Supplementary Tables 1-2

Supplementary Figures 1-11

Supplementary Table 1. Detailed information of the primer positions of the most frequent mutations in the TRs of investigated PCR primer systems.

Mutation	TR (primer system)	Distance from 5' end (bp)	Primer positions at high risk	TR length	Genomic positions range (5'-3')	Effect in PCR
SNP: C21618G	Sarkar-S-F	2	18-22	22	21617-21638	Moderate-risk
SNP: G15451A	Corman-RdRp-F	21	18-22	22	15431-15452	High-risk
SNP: G15451A	Niu-RdRp-F	14	14-18	18	15438-15455	High-risk
SNP: G15451A	Tombuloglu-RdRp-F	13	16-20	20	15439-15458	Moderate-risk
SNP: G15451A	Won-RdRp-1-F	11	16-20	20	15441-15460	Moderate-risk
SNP: G28881T	Niu-N-F	1	18-22	22	28881-28902	Moderate-risk
„AAC"-triplet: G28881A, G28882A, G28883C	Niu-N-F	1-3	l.i.	22	28881-28902	High-risk
Deletion: ATACATG21764A	Young-S-F	2	l.i.	20	21763-21782	High-risk
SNP: C23271A	Davi-S-1-P	24	6-19	24	23248-23271	Moderate-risk
SNP: C23271A	Davi-S-2-P	25	6-20	25	23247-23271	Moderate-risk
SNP: C28977T	Niu-N-R	3	18-22	22	28979-28958	Moderate-risk
SNP: C26270T	Sarkar-E-F	17	17-21	21	26254-26274	High-risk
SNP: C26270T	Corman-E-F	2	22-26	26	26269-26294	Moderate-risk
SNP: C26270T	Mollaei-E-F	9	16-20	20	26262-26281	Moderate-risk
SNP: C26270T	Tombuloglu-E-F	9	18-22	22	26262-26283	Moderate-risk
SNP: C26270T	Won-E-1-F	12	17-21	21	26259-26279	Moderate-risk
SNP: C26270T	Won-E-2-F	12	18-22	22	26259-26280	Moderate-risk
SNP: C28311T	Lu-N-1-probe	3	6-19	24	28309-28332	Moderate-risk

Abbreviations: l.i. - location independent

Supplementary Table 2. Summary of the most frequent mutations in the TRs of investigated PCR primer systems in the GISAID database from 1st Jan 2021 to 31st Mar 2022.

Primer	Mutation	Ratio of mutated samples in the GISAID database	Ratio of mutated samples by WHO designation (*)
Niu-N-F[M]	*SNP:* **G28881T**	48.84%	Delta (99.55%), Other variant (35.09%), Beta (1.74%), Gamma (<1%), Omicron (<1%), Alpha (<1%)
Sarkar-S-F[M]	*SNP:* **C21618G**	48.55%	Delta (99.2%), Other variant (31.53%), Beta (1.72%), Gamma (<1%), Omicron (<1%), Alpha (<1%)
Corman-RdRp-F[H], Niu-RdRp-F[H], Tombuloglu-RdRp-F[M], Won-RdRp-1-F[M]	*SNP:* **G15451A**	48.25%	Delta (98.41%), Other variant (32.96%), Beta (1.8%), Gamma (<1%), Omicron (<1%), Alpha (<1%)
Lu-N-1-probe[M]	*SNP:* **C28311T**	29.23%	Omicron (98.3%), Beta (4.41%), Gamma (2.61%), Other variant (1.6%), Delta (<1%), Alpha (<1%)
Sarkar-E-F[H], Corman-E-F[M], Mollaei-E-F[M] Tombuloglu-E-F[M], Won-E-1-F[M], Won-E-2-F[M]	*SNP:* **C26270T**	29.17%	Omicron (99.15%), Other variant (<1%), Alpha (<1%), Gamma (<1%), Beta (<1%), Delta (<1%)
Won-S-1-R[M]	*SNP:* **C23202A**	22.51%	Omicron (76.68%), Other variant (<1%), Gamma (<1%), Alpha (<1%), Beta (<1%), Delta (<1%)
Chan-S-probe[M]	*SNP:* **G22813T**	22.26%	Omicron (74.04%), Beta (11.0%), Other variant (<1%), Delta (<1%), Gamma (<1%), Alpha (<1%)
Davi-S-1-probe[M], Davi-S-2-probe[M]	*SNP:* **C23271A**	13.15%	Alpha (99.81%), Gamma (44.35%), Other variant (6.58%), Beta (<1%), Delta (<1%), Omicron (<1%)
Niu-N-R[M]	*SNP:* **C28977T**	13.14%	Alpha (99.79%), Gamma (44.7%), Other variant (5.62%), Beta (<1%), Delta (<1%), Omicron (<1%)
Young-S-F[H]	*Deletion:* **ATACATG21764A**	19.33%	Alpha (97.47%), Omicron (9.67%), Other variant (4.14%), Delta (<1%), Beta (<1%), Gamma (<1%)
Niu-N-F[H]	*„AAC"-triplet:* **G28881A, G28882A, G28883C**	44.77 %	Alpha (99.98%), Gamma (99.69%), Omicron (98.95%), Other variant (<1%)

Primer names are based on the nomenclature: [first author last name]-[target gene name]-[id, when multiple primer systems target the same gene]-[type of oligo: forward (F), reverse (R) or probe (P)]. „M" marks the primers where the variant was defined as a moderate-risk mutation; „H" marks the primers if the variant was defined as a high risk mutation. Mutation names are based on the nomenclature: [reference base][genomic position of the start of the variant][alternate non-reference base]. Asterisk: ratio of samples which contain the mutation in a given WHO designation. Lineages with no mutated samples are not listed. Abbreviations: SNP - Single-Nucleotide Polymorphism.

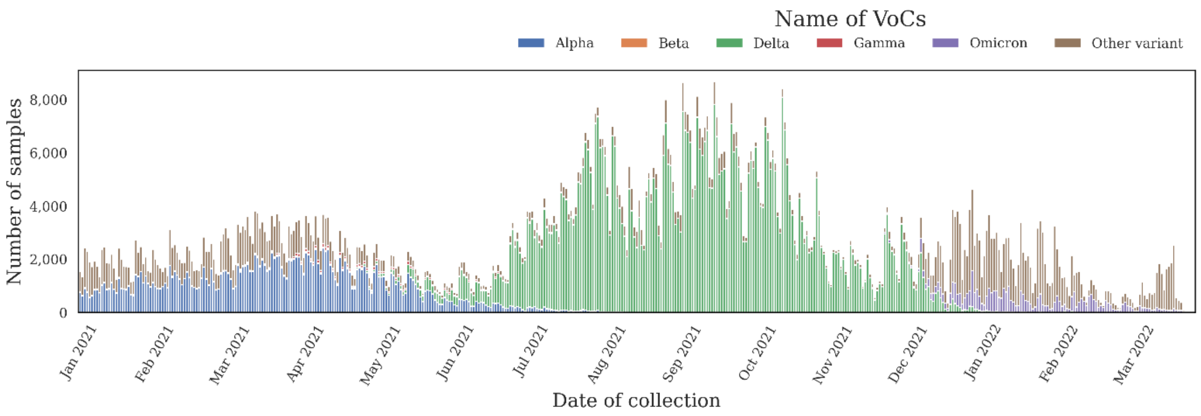

Supplementary Figure 1. Number of daily incoming SARS-CoV-2 samples in the CoVEO database colored by variants.

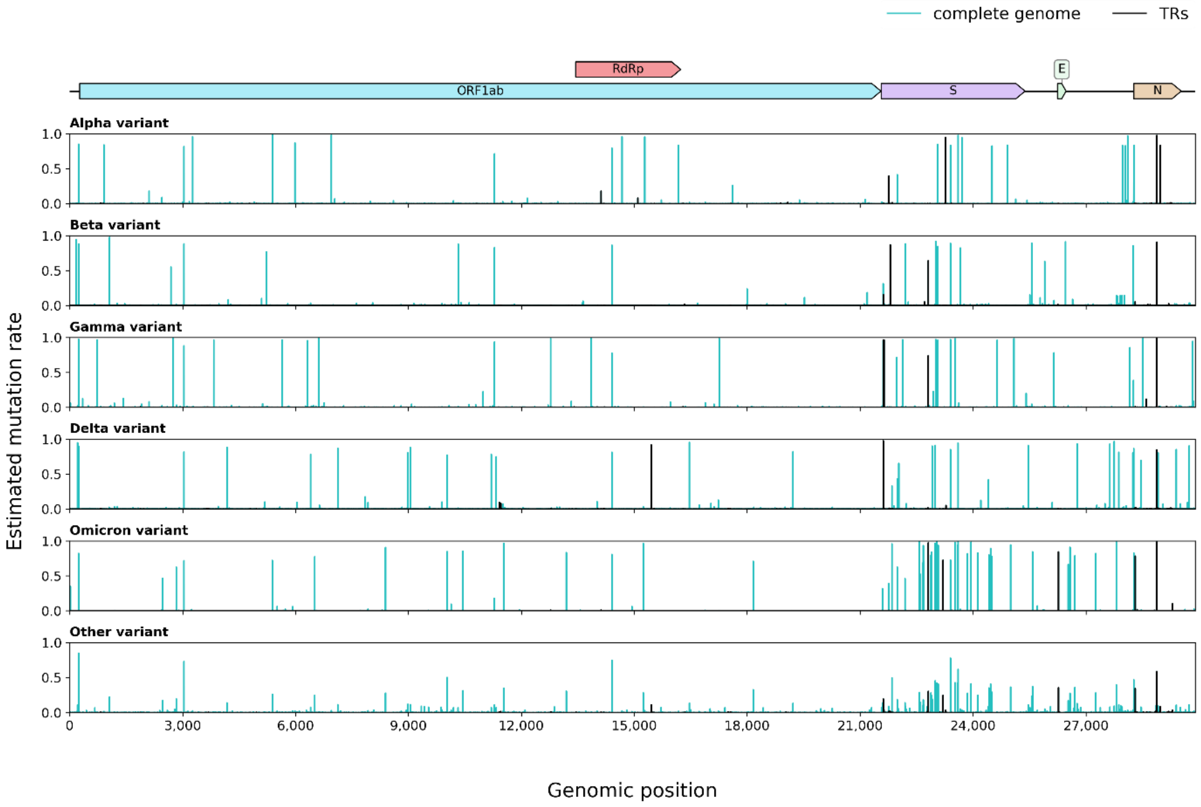

Supplementary Figure 2. Overview of PCR primer TRs and average rate of mutations along the length of the SARS-CoV-2 genome. **Upper panel:** SARS-CoV-2 isolate Wuhan-Hu-1, complete genome (NCBI ID of the fasta sequence: NC_045512) showing genes coding proteins located in ORF1ab (including RdRp), spike protein (S), envelope protein (E), and nucleocapsid protein (N). **Bottom six panels:** Estimated mutation rate of a genomic position in the CoVEO database separately for different variants. For further details, see Methods.

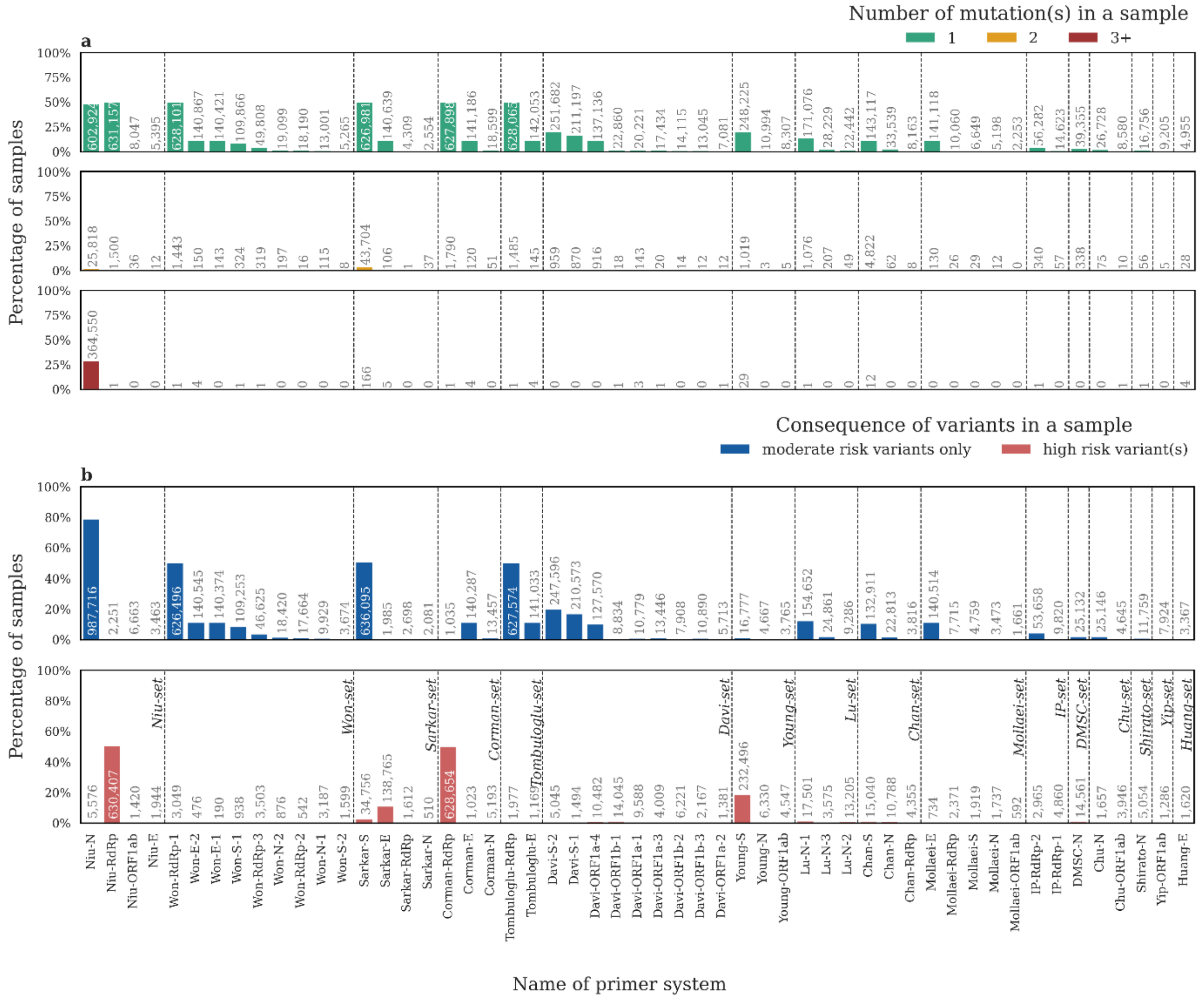

Supplementary Figure 3. Number of mutations and their possible effect on PCR amplification, plotted on a linear scale. **a.** The percentage and number of samples with one (green bars), two (yellow bars) and three or more (red bars) variants in the TRs of different primer systems. **b.** The percentage and number of samples with variants in the TRs of different primer systems. Samples that contain a variant in at least one „high risk" position in the TRs of the given primer system are marked with red, other samples having only „moderate risk" mutations in the given TRs are presented in blue. For further details on mutation classification, see Methods. Primer system names are based on the nomenclature: [first author last name]-[target gene name]-[id, when multiple primer systems target the same gene]. Samples with no variants in the given TRs are not shown.

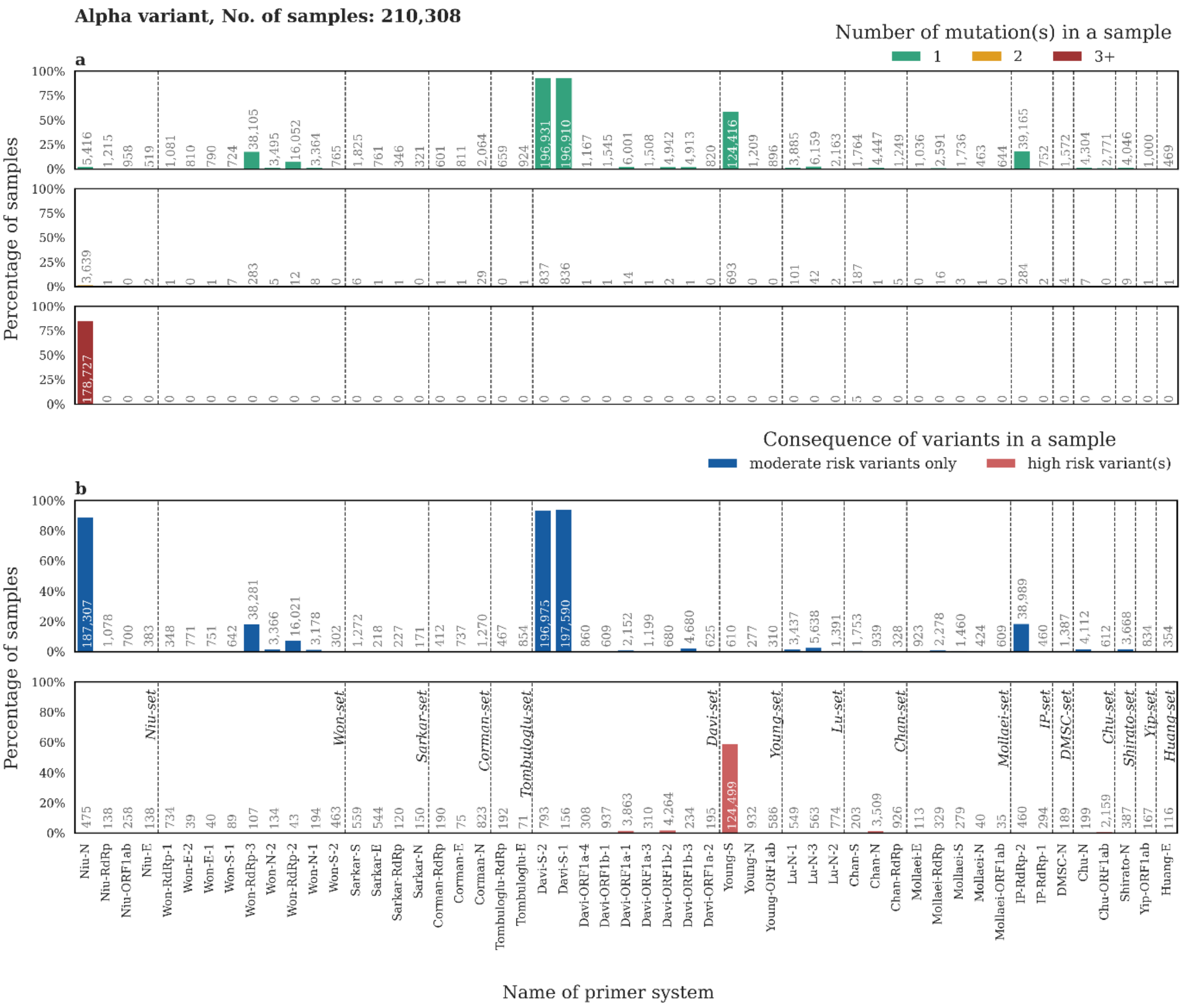

Supplementary Figure 4. Number of mutations and their possible effect on PCR amplification, plotted on a linear scale for samples belonging to the Alpha variant. **a.** The percentage and number of Alpha variant samples with one (green bars), two (yellow bars) and three or more (red bars) variants in the TRs of different primer systems. **b.** The percentage and number of Alpha variant samples with variants in the TRs of different primer systems. Samples that contain a variant in at least one „high risk" position in the TRs of the given primer system are marked with red, other samples having only „moderate risk" mutations in the given TRs are presented in blue. For further details on mutation classification, see Methods. Primer system names are based on the nomenclature: [first author last name]-[target gene name]-[id, when multiple primer systems target the same gene]. Samples with no variants in the given TRs are not shown.

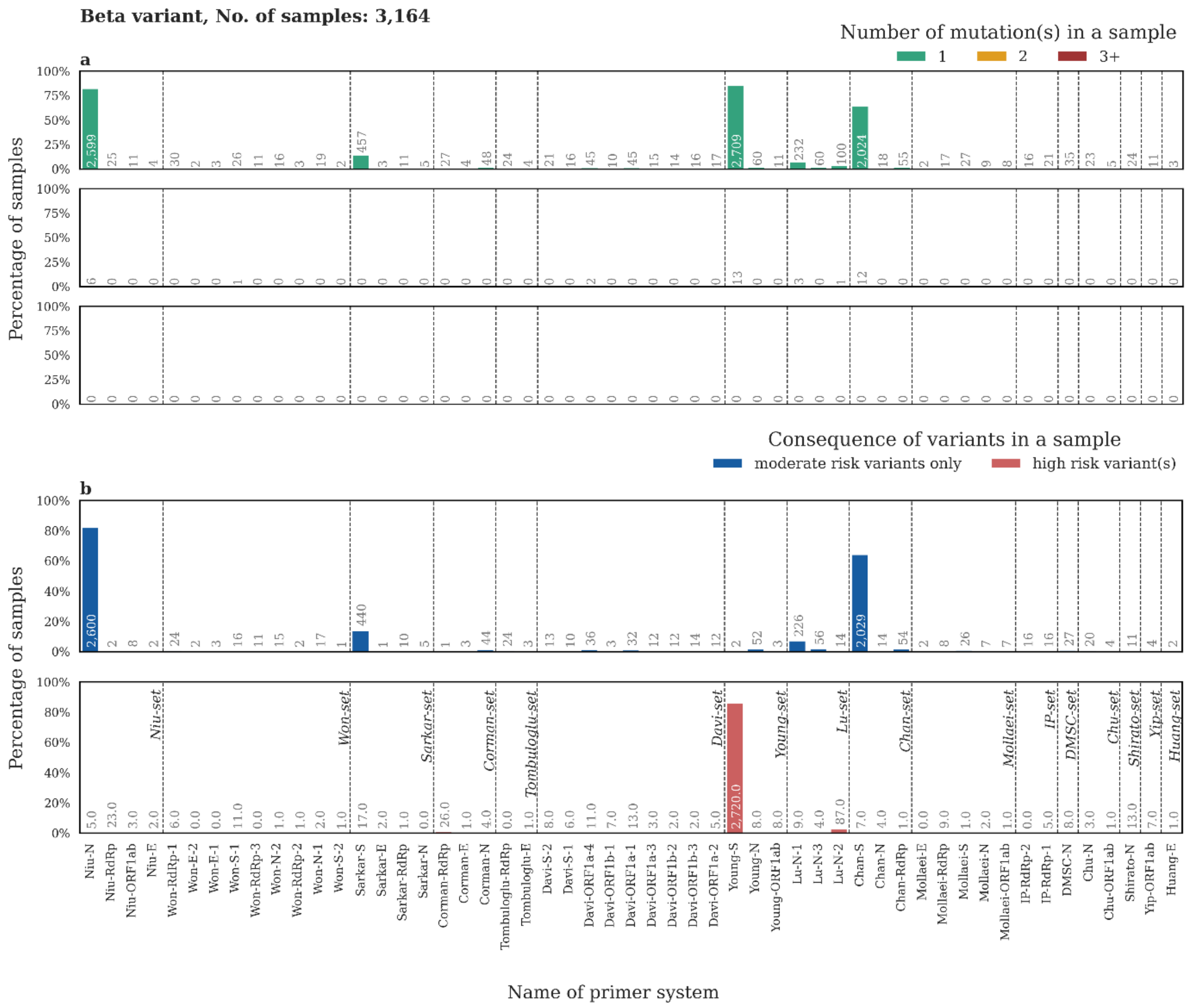

Supplementary Figure 5. Number of mutations and their possible effect on PCR amplification, plotted on a linear scale for samples belonging to the Beta variant. **a.** The percentage and number of Beta variant samples with one (green bars), two (yellow bars) and three or more (red bars) variants in the TRs of different primer systems. **b.** The percentage and number of Beta variant samples with variants in the TRs of different primer systems. Samples that contain a variant in at least one „high risk” position in the TRs of the given primer system are marked with red, other samples having only „moderate risk” mutations in the given TRs are presented in blue. For further details on mutation classification, see Methods. Primer system names are based on the nomenclature: [first author last name]-[target gene name]-[id, when multiple primer systems target the same gene]. Samples with no variants in the given TRs are not shown.

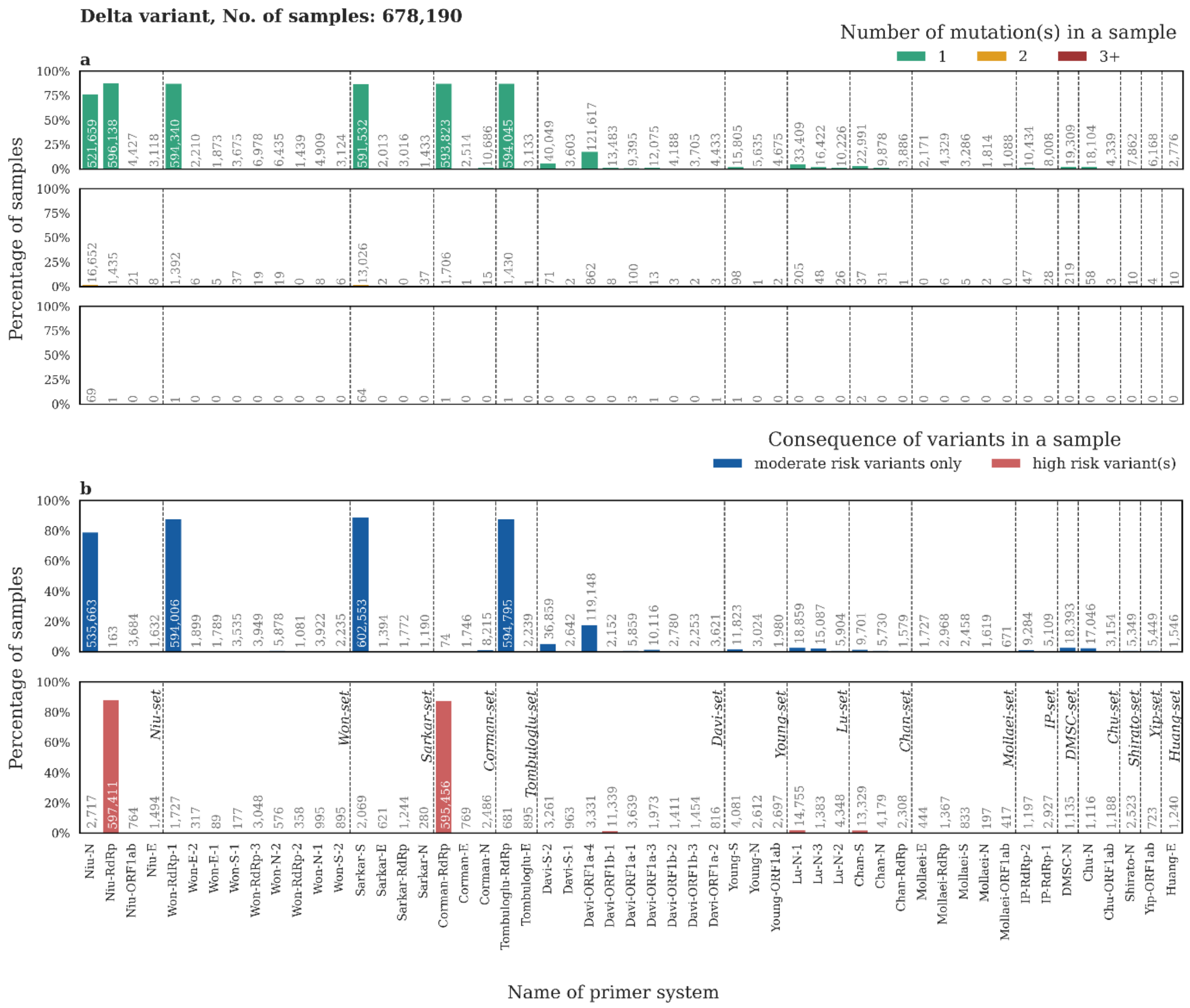

Supplementary Figure 6. Number of mutations and their possible effect on PCR amplification, plotted on a linear scale for samples belonging to the Delta variant. **a.** The percentage and number of Delta variant samples with one (green bars), two (yellow bars) and three or more (red bars) variants in the TRs of different primer systems. **b.** The percentage and number of Delta variant samples with variants in the TRs of different primer systems. Samples that contain a variant in at least one „high risk" position in the TRs of the given primer system are marked with red, other samples having only „moderate risk" mutations in the given TRs are presented in blue. For further details on mutation classification, see Methods. Primer system names are based on the nomenclature: [first author last name]-[target gene name]-[id, when multiple primer systems target the same gene]. Samples with no variants in the given TRs are not shown.

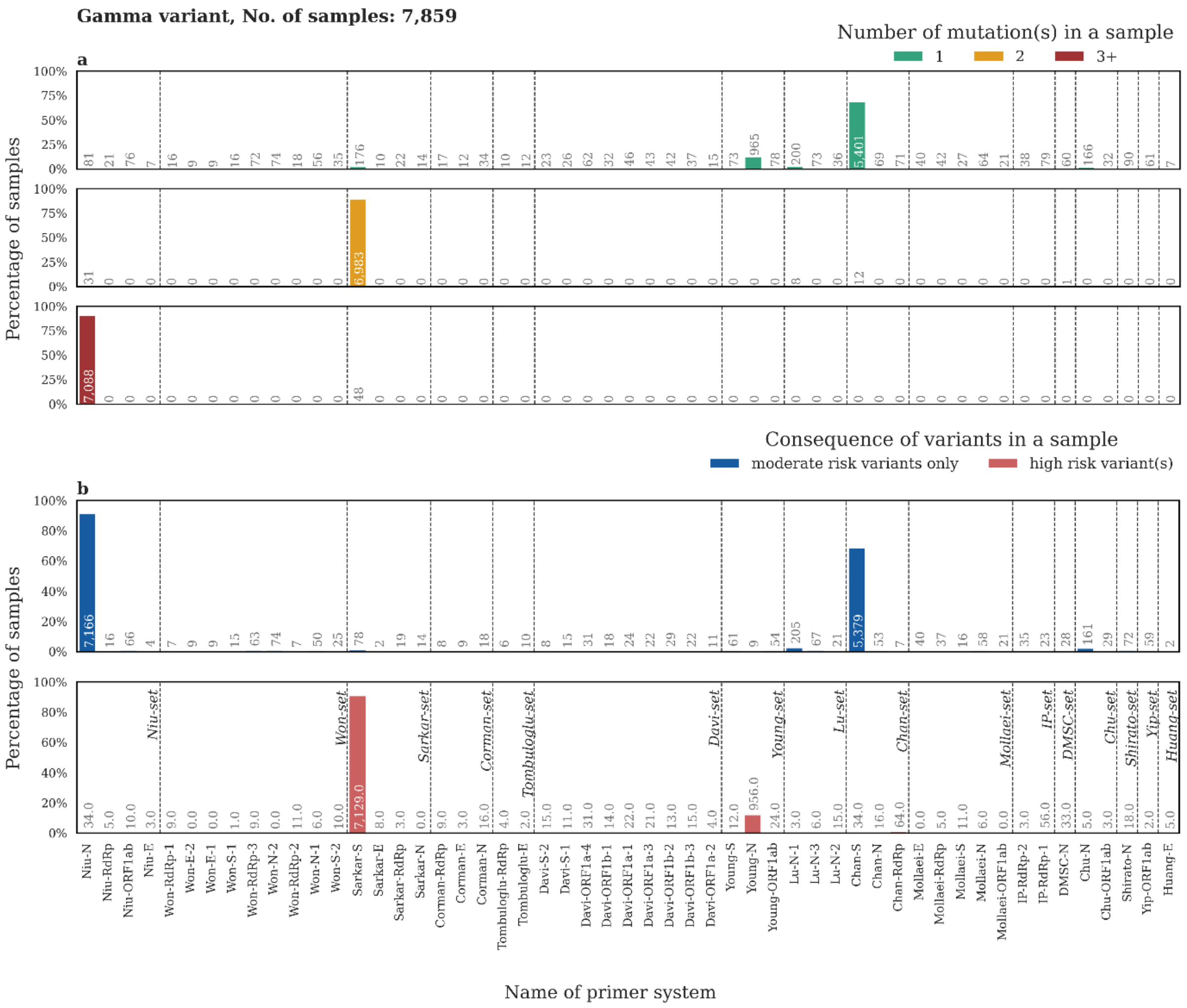

Supplementary Figure 7. Number of mutations and their possible effect on PCR amplification, plotted on a linear scale for samples belonging to the Gamma variant. **a.** The percentage and number of Gamma variant samples with one (green bars), two (yellow bars) and three or more (red bars) variants in the TRs of different primer systems. **b.** The percentage and number of Gamma variant samples with variants in the TRs of different primer systems. Samples that contain a variant in at least one „high risk" position in the TRs of the given primer system are marked with red, other samples having only „moderate risk" mutations in the given TRs are presented in blue. For further details on mutation classification, see Methods. Primer system names are based on the nomenclature: [first author last name]-[target gene name]-[id, when multiple primer systems target the same gene]. Samples with no variants in the given TRs are not shown.

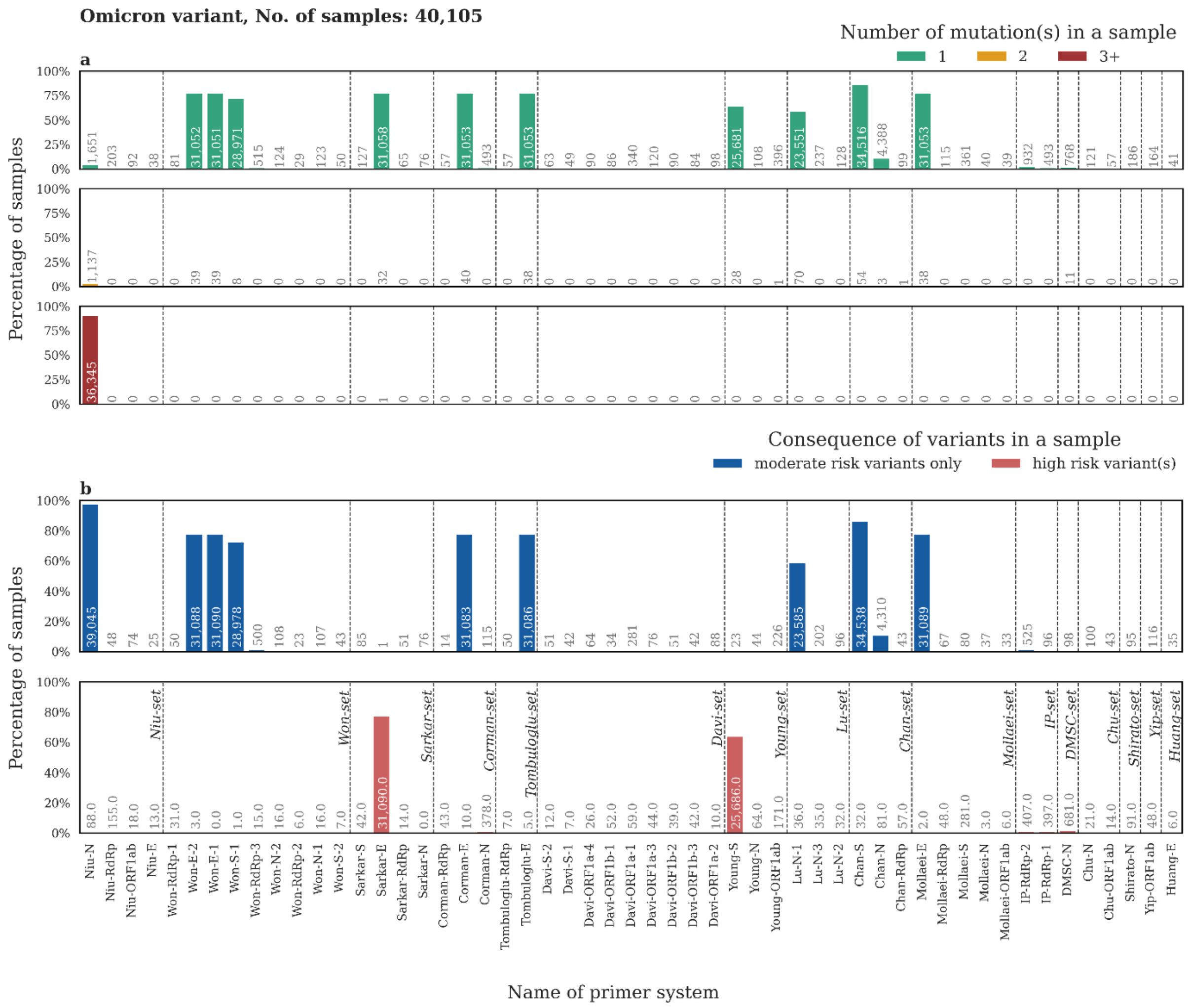

Supplementary Figure 8. Number of mutations and their possible effect on PCR amplification, plotted on a linear scale for samples belonging to the Omicron variant. **a.** The percentage and number of Omicron variant samples with one (green bars), two (yellow bars) and three or more (red bars) variants in the TRs of different primer systems. **b.** The percentage and number of Omicron variant samples with variants in the TRs of different primer systems. Samples that contain a variant in at least one „high risk" position in the TRs of the given primer system are marked with red, other samples having only „moderate risk" mutations in the given TRs are presented in blue. For further details on mutation classification, see Methods. Primer system names are based on the nomenclature: [first author last name]-[target gene name]-[id, when multiple primer systems target the same gene]. Samples with no variants in the given TRs are not shown.

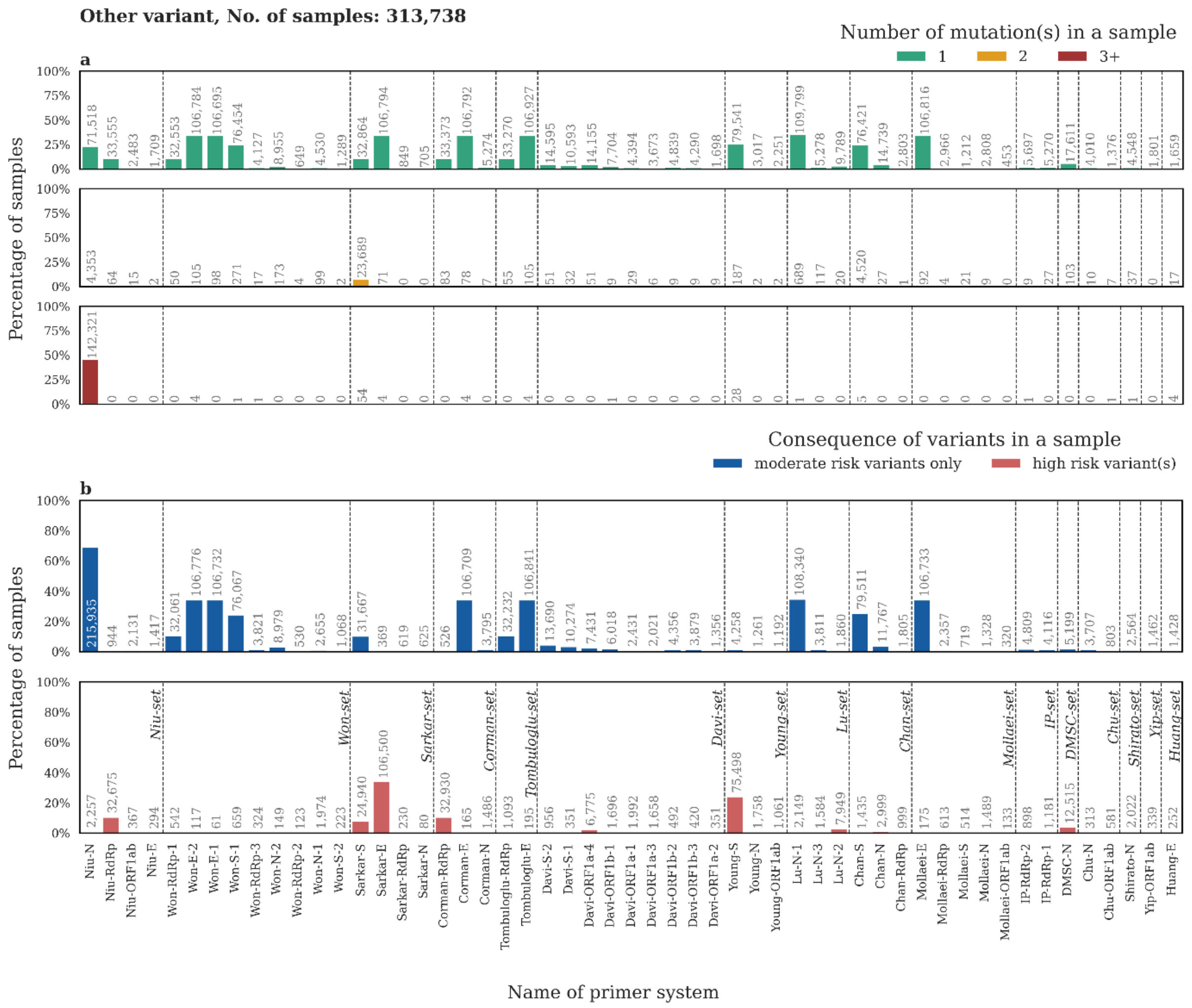

Supplementary Figure 9. Number of mutations and their possible effect on PCR amplification, plotted on a linear scale for samples belonging to the “Other variant” category. **a.** The percentage and number of “Other variant” samples with one (green bars), two (yellow bars) and three or more (red bars) variants in the TRs of different primer systems. **b.** The percentage and number of “Other variant” samples with variants in the TRs of different primer systems. Samples that contain a variant in at least one „high risk” position in the TRs of the given primer system are marked with red, other samples having only „moderate risk” mutations in the given TRs are presented in blue. For further details on mutation classification, see Methods. Primer system names are based on the nomenclature: [first author last name]-[target gene name]-[id, when multiple primer systems target the same gene]. Samples with no variants in the given TRs are not shown.

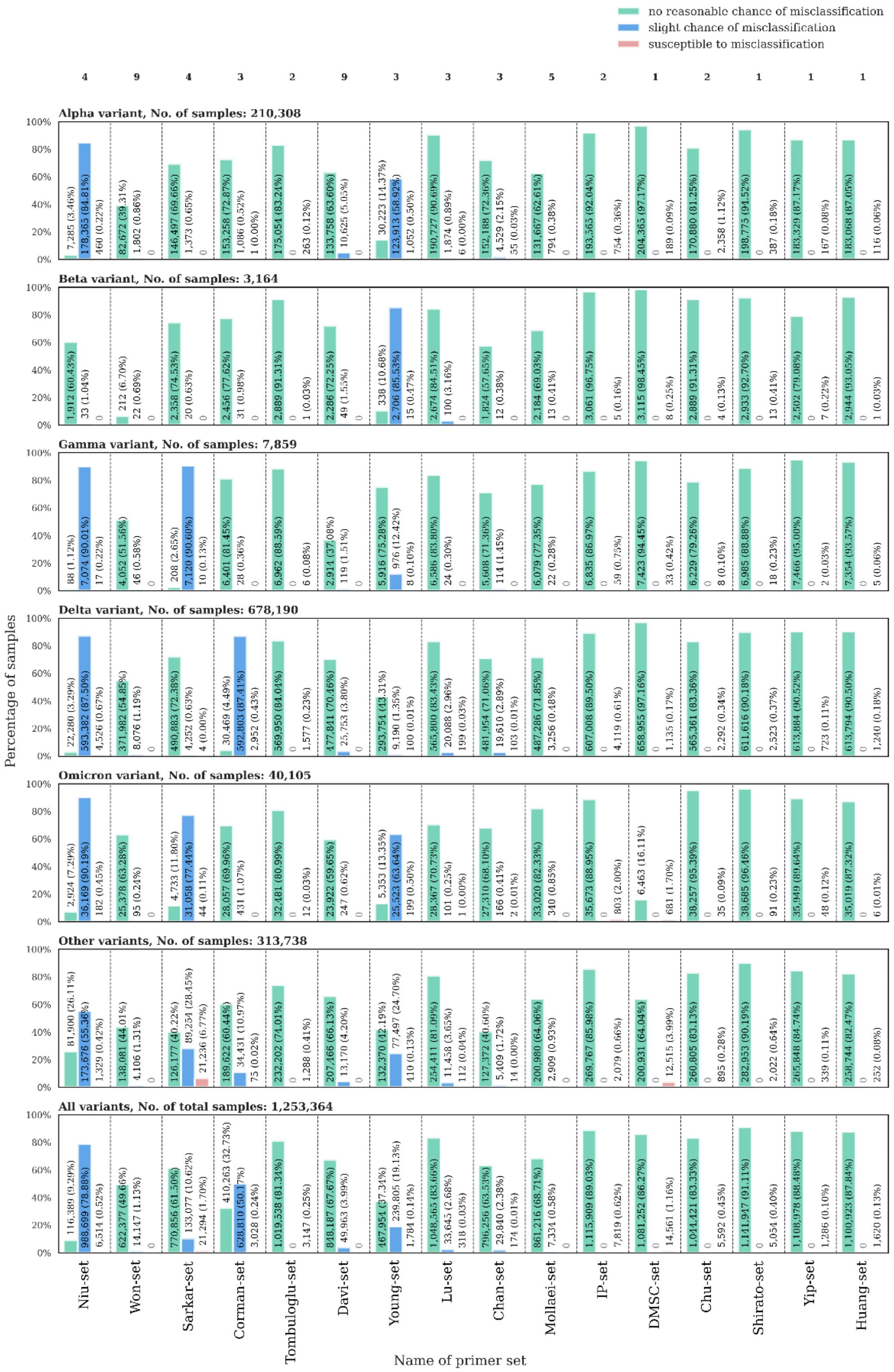

Supplementary Figure 10. Ratio and number of samples having no reasonable chance (light green) or a slight chance of misclassification (light blue) or being susceptible to (light red) misclassification by different primer sets on a linear scale. Numbers on top indicate the number of primer systems present in a given primer set. Primer-set names are based on the nomenclature: [first author last name]-[set]. Ambiguous samples with unsatisfactory coverage in TRs are not shown.

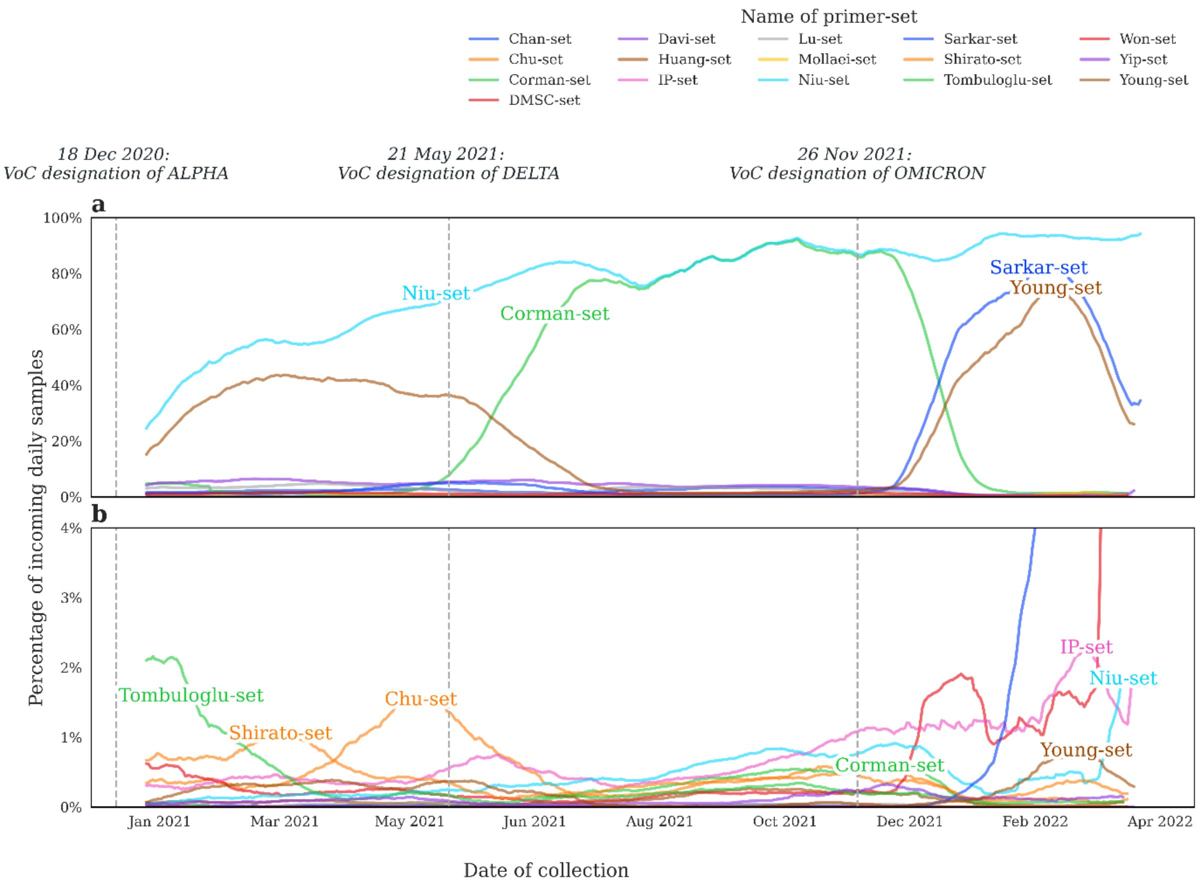

Supplementary Figure 11. Percentage of samples (a) having a slight chance of or (b) being susceptible to misclassification with different primer sets over time (30-day rolling average). (A zoomed-in version of Figure 5.)
